# Legal Relief for Children in Immigrant Families: A Mixed-Methods Study

**DOI:** 10.3390/ijerph19074373

**Published:** 2022-04-05

**Authors:** Natalie Wichelt, Kenny Torres, Gabriela de la Vega, Julie M. Linton, Kimberly Montez

**Affiliations:** 1 Department of Pediatrics, Wake Forest School of Medicine, Medical Center Boulevard, Winston-Salem, NC 27157, USA; nwichelt@wakehealth.edu; 2 Department of Pediatrics, Wake Forest Baptist Health, Downtown Health Plaza Clinic, 1200 N Martin Luther King, Jr Drive, Winston-Salem, NC 27101, USA; ktorres@wakehealth.edu; 3 Office of the Dean of Students, Wake Forest University, 1834 Wake Forest Rd, Winston-Salem, NC 27109, USA; delag19@wfu.edu; 4 Office of Student Affairs and Admissions, University of South Carolina School of Medicine Greenville, Department of Pediatrics, Prisma Health Children’s Hospital Upstate, 607 Grove Rd, Greenville, SC 29605, USA; julie.linton@prismahealth.org

**Keywords:** children in immigrant families, health equity, legal problems, primary care

## Abstract

Objective: Immigration status is a known social driver of health. Little research exists on addressing concerns about immigration status in primary care. The objective of this study is to understand the experiences of immigrant families that received a clinical-community intervention to address immigration-related concerns. Methods: We conducted a mixed-methods study: an embedded experimental design study. We implemented an immigration-focused legal needs screening tool and referral intervention at one academic primary care clinic in January 2018. Caregivers who screened positive for immigration-related concerns and were referred to a local immigration law firm were invited to participate in a semi-structured interview. Interviews were recorded, de-identified, transcribed, and systematically coded using an inductive content analysis approach. A modified constant comparative method was used to iteratively review codes, identify emerging themes, and resolve differences through consensus. Results: Nineteen caregivers reported immigration-related legal concerns, seven of whom were interviewed. Most (84.2%) were mothers, identified as Hispanic (94.7%), were from Mexico (52.6%), and reported persecution and fear of returning to their country of origin (57.9%). In interviews, we identified three major themes: (1) families were motivated to immigrate due to mental health trauma and persecution; (2) families sought legal services for several immigration-related concerns; and (3) families experienced challenges in navigating the legal system, with which clinics may assist. Conclusion: This study demonstrates the need for immigration-related services in primary care settings and the feasibility and potential benefits of implementing a legal screening and referral intervention.

## 1. Introduction

Immigration status is a social driver of health (SDH) that impacts the physical and psychosocial wellbeing and self-actualization of children and families [1,2,3]. An undocumented immigrant is defined as a foreign-born person residing in the United States (US) without legal permission [4]. Undocumented immigrants are more likely to report poorer health status and have decreased access to healthcare compared to their US-born counterparts [5]. Nationally, one in four children lived in immigrant families in 2019, and one in five children lived in immigrant families in North Carolina (NC) [6]. The term “children in immigrant families” (CIF) refers to children under the age of 18 years with at least one immigrant parent who is not a US citizen.

Some CIFs are at risk for separation, including parental detention or deportation, child and family detention, or parent-child separation during arrival to the US. A child experiencing separation from or deportation of a parent or legal guardian is at higher risk of developing mental health problems or behavioral changes due to toxic stress, leading to significant health consequences [7,8,9,10,11,12]. Subsequent immigration proceedings, particularly without legal representation, can exacerbate immigration-related stressors [13,14].

The lack of access to legal counsel can impede an immigrant’s ability to receive a fair hearing. Barriers to accessing legal representation include being confined to jails or federal detention centers, transfers to remote detention centers, and the inability to pay for an attorney [15]. Furthermore, undocumented individuals in immigration proceedings are entitled to representation by attorneys but not at the government’s expense and without the obligation to provide an attorney [16]. North Carolina ranks last in the nation for the likelihood of legal representation in immigration cases, with only 23% having representation [17]. When represented legally, 73% of children under 18 years without lawful status who are not accompanied by a parent or legal guardian are allowed to remain in the US, whereas only 15% of unrepresented children are allowed to stay [15]. Securing legal representation increases the likelihood of being released from detention, appearing in court for subsequent hearings, winning removal cases, and seeking relief from deportation [15,18]. If legal cases are successful, securing lawful status ultimately confers eligibility for health coverage [19].

Medical legal partnerships (MLPs) build integrated healthcare systems to address health harming social needs by leveraging legal expertise to advance health. Because many MLPs receive federal funding from the Legal Services Corporation (LSC, Washington, D.C., USA), which excludes undocumented immigrants from eligibility for services in most cases, many immigration-related concerns do not qualify for referral [20]. Although several practical models exist for immigration-focused MLPs, limited research explores primary care models that address concerns regarding family immigration status [21]. The objective of this mixed-methods study is to understand the experiences of immigrant families that received a clinical-community intervention targeting immigration-related legal problems.

## 2. Materials and Methods

### 2.1. Study Design and Community Partnership

This mixed-methods retrospective cohort study was a partnership between a large health system and a local immigration law firm. We conducted a mixed-methods, embedded experimental design to determine the impact of a clinical-community legal partnership. Quantitative results were used to inform the qualitative study, which occurred after participants had access to resources, to more completely describe the program’s impact. The two datasets were merged during the interpretation phase so that the qualitative results were used to describe the quantitative results [22].

### 2.2. Study Setting and Participants

We implemented a novel legal needs screening tool and referral intervention at one academic primary care clinic in January 2018. The academic primary care clinic serves over 10,000 children annually, primarily from low-income populations. Of this patient population, 92% are Medicaid-insured, and the majority (92%) self-identify as Black or Hispanic. Beginning in January 2018, the clinic began screening all patients and families presenting for any visit type for legal concerns using a paper-based questionnaire in English or Spanish depending on the caregiver’s preferred language, with a video or in-person interpreter available to verbally screen for all other languages. The questionnaire screens for multiple unmet social needs, such as food insecurity. Response options are “yes” or “no” with “yes” indicating a positive screen. The question regarding legal concerns asks, “Does your family need a lawyer to help with your landlord, housing, immigration, or taxes?” For families screening positive, the provider assesses whether the family would like assistance. For families requesting assistance with a landlord, housing or taxes, a referral is made to an MLP supported by the LSC. For families requesting assistance with immigration-related concerns, an additional, novel immigration legal screening tool, administered by a licensed clinical social worker (K.T.) due to the sensitivity of the topic, determines eligibility for referral to a local immigration law firm. If any question on the tool is answered “yes”, families are referred to the local immigration law firm that provides reduced cost consultations paid for by the clinic with grant funding, and at no cost to the family. Families agreeing to legal referral signed a Health Insurance Portability and Accountability Act release of information form, and a legal consultation appointment was scheduled by the licensed clinical social worker (K.T.). All caregivers of pediatric patients who underwent screening with the additional legal screening tool that agreed to a referral were 18 years of age or older and spoke either English or Spanish as their primary language were eligible to be included in this study.

From January 2018 to January 2020, there were 21,071 clinic visits with unmet social needs screening, for which 190 (0.9%) had a positive legal concern screen. Of those 190 visits, 19 reported immigration-related legal concerns, underwent additional evaluation on the same day as the office visit with the immigration legal screening tool, all of which were positive, and received a same-day referral to the local immigration law firm. Beginning in June 2020, all 19 of these families were contacted via telephone for interviews; 10 were unable to be contacted (wrong or disconnected number or no answer after 3 attempts), 2 declined participation, and 7 agreed to participate and were mailed a cover letter with study information. The length of time between the legal referral and interview varied between 9 months and 2 years.

### 2.3. Data Collection

#### 2.3.1. Immigration Legal Screening Tool

The screening tool was developed based on literature and in conjunction with attorneys at the local immigration law firm based on criteria for gaining any type of lawful status [23]. At the time of the visit in which the family screened positive for immigration-related legal concerns, this additional legal screening tool was administered. The immigration legal screening tool consists of 12 items that inquired about a history of persecution, being a victim of abuse, mistreatment, neglect, abandonment, domestic violence, trafficking or a crime, chronic health conditions, parental legal status, deportation proceedings, and whether immigration paperwork had been filed.

#### 2.3.2. Interviews

By conducting a detailed review of the literature [21,24] and consultation with an attorney from the local immigration law firm, an interview guide was developed to elicit caregivers’ reasons for seeking an immigration-related legal referral and perceptions about the legal screening, referral, and consultation processes. The interview guide was pilot tested for face validity. Purposive sampling was utilized based on the inclusion criteria and participant consent. Between June and August 2020, after obtaining verbal informed consent, we conducted 7 open-ended, semi-structured individual interviews using the interview guide via telephone. A certified bilingual researcher (K.T.) trained in qualitative interview techniques conducted all the interviews in the caregiver’s preferred language: English or Spanish. Verbal consent was obtained.

### 2.4. Analysis

#### 2.4.1. Quantitative

Descriptive statistics were utilized to analyze the results of the screening tool.

#### 2.4.2. Qualitative

All interviews were recorded, transcribed, and de-identified. Interviews conducted in Spanish were translated into English by a certified translator. Raw narrative data were entered into Atlas.ti (version 8) software (ATLAS.ti Scientific Software Development GmbH, Berlin, Germany) for data analysis. An inductive content analysis approach was used to code interviews, a technique that systematically describes qualitative data [25]. Codes were developed inductively as the code emerged from the data, and a coding scheme and dictionary were developed from the first three interviews. Two researchers (N.W. and G.d.l.V.) coded each transcript independently and assigned codes to specific comments in each transcript based on the coding scheme. If the two researchers disagreed, they discussed their perspectives and sought consensus or worked with members of the research team (K.M. and K.T.) to come to a resolution. A modified constant comparative method was used to iteratively review codes after each coding session and identify emerging themes. The Wake Forest School of Medicine Institutional Review Board approved this study (IRB#00064446).

## 3. Results

### 3.1. Study Population Characteristics

In total, 19 study participants completed the legal needs screening tool, and 7 of the participants completed an additional semi-structured phone interview. The majority of participants who underwent the legal needs screening tool were mothers (84.2%), self-identified as Hispanic (94.7%), were from Mexico (52.6%), and had a mean age of 37 years (Table 1). The children of the participants were of male majority (73.7%), insured by Medicaid (73.7%), and had a mean age of 8 years. The demographic characteristics of participants who underwent semi-structured interviews was very similar, although the mean age of the children was slightly younger (4.5 years) (Table 2).

### 3.2. Novel Legal Screening Tool

Of the 19 participants who completed the legal screening tool, the majority screened positive for both fear of returning to their own country (57.9%) and persecution or mistreatment in their country of origin (57.9%) (Table 3). The majority also reported having family that was a US citizen or had lawful permanent status (52.6%) (Table 3).

### 3.3. Interviews

Of the original 19 survey participants, 7 participated in a semi-structured phone interview. We identified three major themes with additional subthemes. We provide representative quotations for these themes below, with additional supporting quotations (Table 4).

### 3.4. Families Were Motivated to Immigrate Due to Mental Health Trauma and Persecution

In almost all interviews, participants reported that their motivation for immigrating was due to traumatic experiences and/or the fear of hostile treatment. We identified two subthemes within this primary theme.

### 3.5. Mental Health Trauma of Parent or Child Was a Motivator for Immigration

A recurring theme throughout our interviews included deeply distressing experiences prior to immigrating. Trauma served as a motivating factor for participants to leave their country of origin. One mother discussed being a target of violence as her reason for needing to leave her country, stating, “I couldn’t go to my house because one time I received a message that told me what clothes I was wearing, where I was, what I was doing. [The gang] had me under control.” Several mothers discussed how intimate partner violence left them with no other option but to leave their homes. One mother described, “[My daughter’s father] beat me because he was a womanizer. He said I was his servant. He nearly killed me.”. Another participant described, “[My daughter’s father] threatened that he was going to kill my family if I didn’t get back with him, and one time he came into the house and abused me.” Participants recounted witnessing random acts of violence as a motivating factor leaving their country. One participant noted, “I had seen how [the gang] had killed a guy on the side of street. I was going to wait for the bus, and the guy was waiting there at the bus stop too. They shot him, and I had to watch that happen.” Traumatic experiences continued to have lasting mental health effects on participants after immigrating to the US. When discussing the sources of her anxiety and depression, one participant reported, “Just recently I told my child that their father had raped me.”

### 3.6. Escaping Persecution Played a Role in Immigrating

Many participants discussed the role persecution played in immigration, including political affiliation, gang violence, and racial differences. One mother expressed fear for her own safety; she said, “I fled from my country because my brothers were killed.” Another discussed, “My concern is that the father of my children was murdered in Mexico by drug traffickers.” Yet another expressed apprehension about a threat to her child’s life; she stated, “To tell you the truth, I was scared that they were going to kill [my daughter]. The drug traffickers were already following her.” Several discussed safety concerns and that immigration was the only option to protect their family.

### 3.7. Families Sought Legal Services for a Variety of Immigration-Related Concerns

The purpose for families seeking legal services largely centered on immigration-related concerns about citizenship, legal status, and the Deferred Action for Childhood Arrivals (DACA) program. The fear of deportation commonly served as a strong motivation for pursuing legal representation. This worry was most often experienced by the caregiver for the family. For example, one participant stated, “I was looking for an attorney because if the lawyer did not represent me, I automatically had a deportation order.”

For other participants, reasons for seeking a lawyer were due to DACA or a desire to obtain legal status. One participant stated, “I really went to this lawyer to see if I could become a citizen or a resident, and he just basically told me I was unable to get DACA.” Another participant reported the need to obtain legal permission to stay in the US; she stated, “The problem is, my daughter’s permit expired, and the lawyer hasn’t submitted a new permit. She is without a social [security number] and without a permit.”

The challenges of obtaining legal status were not exclusive to immigrants from Central or South America. For instance, one participant reported, “We’re having difficulty getting my son’s paperwork from Canada. He was adopted in Canada. We are having difficulties getting his actual immigration papers here for him to be legal here.”

Lastly, some participants expressed that their motivation for immigrating and pursuing legal processes revolved around a hope to provide their children a better life. “I made the decision to bring my child [to the US] because he was born a little special. My child will have a better opportunity, a better education, and better growth [here]. And if it’s God’s will, then they will treat him so that he will improve.”

### 3.8. Immigrant Families Experienced Challenges in Navigating the Legal System, with Which Clinics May Assist

Navigating the legal system presented a variety of challenges for participants, including finances and distrust in the legal system, which are two subthemes within the primary theme. However, participants provided feedback regarding their experience with the clinic-based legal screening and referral system, which is another subtheme.

### 3.9. Financial Limitations Presented Barriers to Receiving Legal Services

Financial strain and expensive legal fees were factors that prevented individuals the most from securing a legal remedy and created frustration. Furthermore, participants described experiences in which they felt they were being taken advantage of or did not receive the legal help that they needed: “The only detail is that he clearly told me that the child could easily gain residency status, but he didn’t recommend anything for me. Instead, he then asked me for three thousand dollars. It was six thousand that I was going to be charged.” Upon inquiring about the financial difficulties, she explained, “At that time I wasn’t working. I was living with a friend, and what little I earned wasn’t even enough to make ends meet. I couldn’t give [the lawyer] the amount of money he was asking of me. I didn’t have a choice.” Another participant described a negative experience with a previous lawyer; she noted, “He did help us; [he] guided us in the right direction, but at this point the other lawyer had taken all of our money, so we didn’t have extra money to give him for him to actually to do the case for us.”

### 3.10. Distrust in the Immigration Legal System Caused Frustration

For many, the distrust of the legal system was rooted in years of trying to unsuccessfully obtain immigration-related assistance and frustration with waiting so long for the legal processes to occur. “I have been going to immigration court for 6 years.” Another participant reported that the fear of deportation and past negative experiences with lawyers were contributing factors to general distrust of the legal system. Many participants described a convoluted process that did not provide clear solutions or routes to a legal status: “I was apprehended [by ICE]. I escaped. I turned myself into immigration; from there, ICE let me go, and they sent me here.” Another participant described her frustration with the process; she reported, “I don’t have any legal papers for my son so that he can be here, [anything] to be able to win the case, or something to help me to be able to say that yes, they’re going to give me help, and they won’t deport me to my country.”

### 3.11. Participants Had Positive Feedback about the Clinic Experience

Participants were asked to provide their thoughts on the legal screening and referral process at the clinic. Regarding resource provision, one participant said, “The sheet that they gave me with the phone number of the lawyers was good.” Another explained, “I think that the way the form is, is fine, because when someone needs it, they are able to read it and see that you also offer immigration support.”

Participants described the clinical referral to and the meeting with the immigration lawyer as helpful overall. One participant reported, “He explained a lot of stuff to me, so it was actually great. He told me that with me having my CNA [Certified Nursing Assistant] certification, he would probably get me approved.” Another described, “[The lawyer] was really relatable. He was just really nice. Like I said, to me I felt like he went above and beyond when he did the research and met up with us the second time to explain to us what he had found.”

## 4. Discussion

This mixed-methods study evaluated the experiences of immigrant families that received a clinical-community intervention targeting immigration-related legal problems. We found that immigrant families fled their home countries most commonly due to traumatic experiences and persecution. After immigrating, they sought legal services to assist with their legal status but experienced multiple barriers in accessing or receiving help due to finances and distrust. Overall, immigrant families had positive feedback about the clinic experience with legal assistance.

There is evidence to support that immigration status is an important component of SDH given the multiple barriers to accessing and utilizing medical, legal, and social services experienced by immigrant families [1,26]. Therefore, a streamlined screening process and referral model from primary care to legal services could be crucial to improving overall health outcomes for children in immigrant families. While legal assistance is an important consideration for children in immigrant families when examining SDH, there is little logistical guidance on how to facilitate clinical-community legal partnerships in an outpatient setting outside of MLPs that do not address legal status. Our study indicates the feasibility of the following: (1) implementing a novel immigration-based legal needs screening tool and (2) establishing a referral to community-based legal representation.

Previous literature acknowledges the need for immigration-specific legal needs screening [27,28], and our study suggests the feasibility and utility of embedding a novel legal needs screening tool based on the likelihood of meeting legal criteria for gaining lawful status, which is an important factor in determining utility of legal referral. Furthermore, participants found the referral and meeting with an immigration lawyer helpful and provided positive feedback about the clinic model. One study highlighted the potential need for immigration-related services among adults in primary care by utilizing legal screening and a referral to a clinical navigator but demonstrated difficulty in securing legal assistance even after maximizing community resources [24]. Our study suggests a benefit of a direct clinical-community legal partnership, which may decrease barriers in access to legal representation.

In addition to providing an example of a clinical-community legal model, we gained valuable insight into the motivations for immigration, interactions with the legal system and family perspectives, which further add to the existing literature and may influence future clinical interventions. For example, most participants in our study reported traumatic experiences and fear of persecution, indicating that many have the right to pursue international protection that may result in obtaining legal statuses. This finding was consistent with the prior literature, in which most families pass the “credible fear” test, and the majority of children fleeing their home country report facing personal harm and violence [29,30].

Furthermore, our study illuminates the challenges immigrant families face in navigating the legal process and ways that clinical partnerships could be helpful. In a study examining the role of MLPs among immigrants, similar feelings of increased urgency and demand were demonstrated in qualitative interviews [27]. However, the majority of the literature focuses on MLPs and less on the motivating factors and legal barriers, as shown among our study participant interviews, highlighting the value of our study in contributing to the literature [27]. While clinic-based legal interventions targeting immigration status show promise, without a pathway to citizenship or expanded public health insurance eligibility for non-citizens and undocumented immigrants, the impact of these legal interventions on health may be limited.

There are several limitations to our study. First, all participants were recruited from one primary care clinic and referred to a single law firm, so the results may not be generalizable to other institutions. Second, the sample size was limited by those eligible for the study and who agreed to participate; therefore, we were unable to verify whether thematic saturation was reached, although the data from the transcripts were redundant [31]. The length of time between the legal referral and interview may have contributed to an inability to contact the majority of participants. Third, the participants of the study may not be representative of all immigrant families. Fourth, we may have missed immigrant families in need of legal assistance that did not screen positive on the initial social needs form given the lack of validity of the screening question and/or fear and uncertainty regarding affirmative responses.

## 5. Conclusions

Family immigration status is deeply intertwined with the health and wellbeing of immigrant families. For clinics serving large immigrant populations, our study highlighted the importance of addressing immigration-related legal problems in outpatient pediatric settings. Our study also indicated the feasibility and potential benefit of having clinic-based an immigration-based legal needs screening tool and a referral to community-based legal representation. Future research studies are necessary to determine the validity of such a screening tool and expand effective clinic-based legal interventions that address immigration legal status of immigrant families.

## Figures and Tables

**Table 1:** Legal Needs Screening Participant Characteristics.

Study Participants (N = 19)	N (%) or Mean (Range)
Parent/guardian	
Mother	16 (84.2)
Father	3 (15.8)
Parent/guardian age, years	37 (21–58)
Parent/guardian race	
Non-Hispanic Black	1 (5.3)
Hispanic	18 (94.7)
Child age, years	8 (0–19)
Child gender	
Female	5 (26.3%)
Male	14 (73.7)
Preferred Language	
English	2 (10.5)
Spanish	17 (89.5)
Insurance Coverage	
Medicaid	14 (73.7)
Uninsured	5 (26.3)
Country of Origin	
Canada	1 (5.3)
Cuba	1 (5.3)
El Salvador	4 (21.0)
Honduras	3 (15.8)
Mexico	10 (52.6)

**Table 2:** Interview Participants’ Characteristics.

Interview Participants (N = 7)	N (%) or Mean (Range)
Parent/guardian	
Mother	6 (85.7)
Father	1 (14.3)
Parent/guardian age, years	34.4 (21–47)
Parent/guardian race	
Non-Hispanic Black	1 (14.3)
Hispanic	6 (85.7)
Child age, years	4.5 (0–15)
Child gender	
Female	2 (28.6)
Male	5 (71.4)
Preferred Language	
English	2 (28.6)
Spanish	5 (71.4)
Insurance Coverage	
Medicaid	5 (71.4)
Uninsured	2 (28.6)
Country of Origin	
Canada	1 (14.3)
El Salvador	1 (14.3)
Honduras	1 (14.3)
Mexico	4 (57.1)

**Table 3:** Legal Needs Screening Tool Results.

Study Participants (N = 19)	N (%)
Q1. Is the parent or the child in deportation proceedings or has the parent or the child ever been stopped by ICE?	8 (42.1%)
Q2. Does the child or a sibling have significant/chronic health problems?	7 (36.8%)
Q3. Has anyone ever filed immigration paperwork for the parent or child?	4 (21.1%)
Q4. Are one or both of the child’s parents absent or uninvolved in the child’s life?	8 (42.1%)
Q5. Is the child or the parent afraid to return to their home country because they will be persecuted or have been persecuted because of their political opinion, religion, race, nationality, or because of who they are? (Fear of returning to their native country because of the poverty or generalized crime is not enough)	11 (57.9%)
Q6. Has the child been abused, abandoned or neglected by a legal or biological parent?	8 (42.1%)
Q7. Has the child or a parent been victim of a crime, including domestic violence?	7 (36.8%)
Q8. Does the child or a parent have any US citizen or lawful permanent family (parents, spouses, children older than 16)?	10 (52.6%)
Q9. Was the child or a parent mistreated on their journey to the United States?	5 (26.3%)
Q10. Was the child or a parent forced to work on their journey here or after they arrived?	0 (0%)
Q11. Was the child or a parent persecuted, threatened, or mistreated in their country of origin?	11 (57.9%)
Q12. Has the parent of the child been a victim of abuse or mistreatment by a spouse?	5(26.3%)

**Table 4:** Themes, Subthemes, and Representative Quotations.

Theme	Selected Subthemes	Selected Illustrative Quotes
Families were motivated to immigrate due to mental health trauma and persecution	Mental Health Trauma of Parent or Child was a Motivator for Immigration	“I was psychologically manipulated as well. Not just with hitting [physically] but psychologically as well. Sometimes I felt desperate.”
		“I have also had depression and all that. I want us to be [in the US] together peacefully but sometimes [my daughter] cuts herself off from the world. She doesn’t go out, or sometimes she doesn’t even get out of bed. Sometimes she hits the other kids. She feels that maybe it would have been better if she wasn’t born or that it would have been better if I miscarried her before she was born, and well that hurts me; it hurts me a lot.”
		“I was assaulted by the father of my daughter who would hit me and harass me. He ran over me with a car, and yet he was the one who spoke with the police.”
	Persecution Played a Role in Immigrating	“I had to leave for a neighboring city to a friend’s [house]. I don’t know how [the gang] knew where this friend was hiding me. They arrived one night, and they shot two or three times in the air. I then called the police because my children were young, very scared, crying. So now it was an imminent threat. Later I also received quite a few threats.”
		“In the case of the children’s father, I wanted to file a lawsuit, because he was threatening that he was going to take away my daughter. Financially [her father] was fine there. He could buy off the lawyers and he could buy off, if he wanted, the judge to take custody of the child.”
Families Sought Legal Services for a Variety of Immigration-related Concerns		“I want identification. Imagine, I don’t even have an ID here, not even permit or anything because the only identification I have is from Honduras and the one from Honduras is no longer valid.”
		“I was the victim of a crime in which they robed my house. I wanted to ask [the lawyer], because they told me that I could get a worker’s permit, the U Visa, because of this crime so for that I wanted help.”
		“Right now, I’m worried since all of this is going on, and they still haven’t put a stable process in DACA so I would maybe look forward to seeing how I can basically get permanent status where I won’t have to worry about it. I want to continue school and stuff but with me being unemployed and not being from here it’s hard for me to go back to school because everybody charges out of state.”
		“I brought [my daughter] here for asylum but they didn’t permit her through. I did everything possible so they would give her asylum, but no. Maybe the immigration [police] don’t realize who in reality really needs [asylum], and they give support to someone who doesn’t need it, instead of the person who is fleeing [persecution].”
Immigrant Families Experienced Challenges in Navigating the Legal System, With Which Clinics May Assist	Financial Limitations Presented Barriers to Receiving Legal Services	“The only problem was that I needed the help with benefits. I’m not working, and I’m a single mom, and the lawyer was charging me $600 to complete my DACA process. And I’m telling you [I have] no job; I’m unemployed with a child, so I couldn’t really afford it but other than that it was actually great.”
		“So, justifiably, we had to practically sell what little we had to come here. At that time when I came, no one wanted to lend me the money so I that could come [here], not even the bank. So, I had to pawn a piece of land, for which I didn’t get much.”
		“I met with [the lawyer] at the consultation, and then he did some research for us. We met a second time but then that’s when we just had to try doing the paperwork on our own because of the cost at that point.”
	Distrust in the Immigration Legal System Caused Frustration	“We originally had hired a lawyer that ended up basically just taking our money and didn’t do anything for us. So, because of our prior issues that we had with our first lawyer that took forever to try to do paperwork and sucked the money out of our wallets.”
		“[The lawyer] told me that we should go to a psychologist, and maybe with that it would be possible that they could sign the [visa] forms. But it still hasn’t been done. Well, I don’t know if really the lawyer is good for that or not; I don’t know.”
		“Well, the experience was very unpleasant because he didn’t pay attention to me. He reviewed my case. He told me I had a deportation order. I mean, since I [presented to the border], I was automatically taken by ICE without a deportation order. Finally, I told him that what I needed was for him to help me with a [work] permit. He said yes that the permit would come in six months and that I would be charged $1800. Then I thought [the price] was too high and did not see that he was a trustful person; he was a person that… Sometimes there are people who, when you talk, you see their interest [only in the case]. And actually, the lawyer who saw me wasn’t interested in my case.”
	Participants Had Positive Feedback about the Clinic Experience	“The referral to the immigration lawyer was very useful.”
		“Well, I think that the form is fine, because when someone needs it, they are able to read it and see that you also offer immigration support.”
		“Honestly, [the referral] was very helpful, and if I can ask for resources to pay for [legal services], I’ll pay for it so that my child can have his residency.”
		“We appreciated that for free [the lawyer] went and got us in the right direction.”

## Data Availability

The data presented in this study are available upon request from the corresponding author. The data are not publicly available due to privacy restrictions.
